# Anti-RANKL Therapy Prevents Glucocorticoid-Induced Bone Loss and Promotes Muscle Function in a Mouse Model of Duchenne Muscular Dystrophy

**DOI:** 10.1007/s00223-023-01116-w

**Published:** 2023-07-20

**Authors:** Soher Nagi Jayash, Dounia Hamoudi, Louise A. Stephen, Anteneh Argaw, Carmen Huesa, Shuko Joseph, Sze Choong Wong, Jérôme Frenette, Colin Farquharson

**Affiliations:** 1 The Roslin Institute and Royal (Dick) School of Veterinary Studies, University of Edinburgh, Easter Bush, Midlothian, EH25 9RG UK; 2 Centre de Recherche du Centre Hospitalier, Universitaire de Québec-Centre, Hospitalier de L’Université Laval, Université Laval, Quebec City, QC Canada; 3 School of Infection and Immunity, University of Glasgow, Glasgow, UK; 4 Royal Hospital for Children Glasgow, School of Medicine, Dentistry and Nursing, Child Health, Queen Elizabeth University Hospital, Glasgow, UK; 5 University of Glasgow/Royal Hospital for Children Glasgow, School of Medicine, Dentistry & Nursing, Child Health, Queen Elizabeth University Hospital, Glasgow, UK

**Keywords:** Anti-RANKL, Glucocorticoid, Bone loss, Muscle dysfunction, Duchenne muscular dystrophy, Bisphosphonates

## Abstract

Bisphosphonates prevent bone loss in glucocorticoid (GC)-treated boys with Duchenne muscular dystrophy (DMD) and are recommended as standard of care. Targeting receptor activator of nuclear factor kappa-B ligand (RANKL) may have advantages in DMD by ameliorating dystrophic skeletal muscle function in addition to their bone anti-resorptive properties. However, the potential effects of anti-RANKL treatment upon discontinuation in GC-induced animal models of DMD are unknown and need further investigation prior to exploration in the clinical research setting. In the first study, the effects of anti-RANKL and deflazacort (DFZ) on dystrophic skeletal muscle function and bone microstructure were assessed in *mdx* mice treated with DFZ or anti-RANKL, or both for 8 weeks. Anti-RANKL and DFZ improved grip force performance of *mdx* mice but an additive effect was not noted. However, anti-RANKL but not DFZ improved ex vivo contractile properties of dystrophic muscles. This functional improvement was associated with a reduction in muscle damage and fibrosis, and inflammatory cell number. Anti-RANKL treatment, with or without DFZ, also improved trabecular bone structure of *mdx* mice. In a second study, intravenous zoledronate (Zol) administration (1 or 2 doses) following 2 months of discontinuation of anti-RANKL treatment was mostly required to record an improvement in bone microarchitecture and biomechanical properties in DFZ-treated *mdx* mice. In conclusion, the ability of anti-RANKL therapy to restore muscle function has profound implications for DMD patients as it offers the possibility of improving skeletal muscle function without the steroid-related skeletal side effects.

**Supplementary Information:**

The online version contains supplementary material available at 10.1007/s00223-023-01116-w.

## Introduction

Duchenne muscular dystrophy (DMD) is a rare X-linked recessive degenerative muscle condition affecting 1 in 4000 male live births and caused by mutations in the dystrophin-encoding DMD gene [1, 2]. It is often diagnosed in early childhood (around 4 years of age) and without treatment, boys at about 10–11 years of age lose the ability to walk. By their mid-teens, affected boys without treatment develop severe scoliosis, respiratory, and cardiac failure, and the mean age of death without treatment is 19 years of age [3]. While there is no curative therapy, the current standard of care includes the use of glucocorticoids (GC) to slow muscle wasting thereby prolonging age at loss of ambulation by about 2–3 years [2, 4]. GC treatment has also been shown to be beneficial for respiratory, cardiac status, and upper limb function in these boys [5].

Long-term use of GCs is, however, associated with significant side effects in particular bone morbidity leading to osteoporotic fractures [6]. GCs promote osteoclast formation and activity by increasing receptor activator of nuclear factor kappa-B ligand (RANKL) production by osteoblasts and osteocytes and down-regulating its soluble decoy receptor osteoprotegerin (OPG) [7]. This skews the RANKL:OPG ratio toward osteoclastogenesis [8–10]. In addition, despite the use of GC therapy, muscle function inevitably deteriorates and the majority of patients become non-ambulant by early adolescence. As much of the mechanical stimuli detected by osteocytes originates from muscle contraction forces, muscle wasting leads to the loss of cross-talk between muscle and bone [11]. This cross-talk is essential for the structural maintenance of bones [12]. Therefore, DMD is a model of a persistent, irreversible, and progressively worsening threat to bone health-driven largely by the use of GC but compounded by the dystrophic muscle process [13].

Anti-resorptive medications such as bisphosphonates, which inhibit osteoclast activity, are in accordance with current international standards of care considered a first-line treatment to prevent further bone loss in DMD following identification of fractures [14]. This is administered by intravenous infusions and side effects especially following first infusion are common include fever, muscle aches, nausea, and vomiting, which can be clinically significant on the background of adrenal insufficiency in the context of long-term use of GC in this population. Rarer side effects in DMD include rhabdomyolysis have been reported [14]. Therefore, alternative bone protective medicines are required that are effective, easily administered, and have minimal side effects.

Denosumab is a biological anti-resorptive therapy that binds to RANKL inhibiting the formation, function, and survival of osteoclasts [15]. The potential benefit of denosumab over bisphosphonates in the treatment of GC-induced osteoporosis (GIO) was demonstrated in adults receiving long-term GCs who were switched from oral bisphosphonates to denosumab [16]. After treatment for 12 months, there was a greater increase in bone mineral density and improved suppression of bone turnover [16]. In addition to its anti-resorptive role, evidence is now accumulating that the RANKL/RANK/OPG pathway can also influence muscle function by modulating pro-inflammatory genes via the transcription factor, nuclear factor kappa-B (NF-κB) [17]. Specifically, both RANK and RANKL protein levels are increased in the microenvironment of dystrophic myofibres and mice with a muscle-specific RANK deletion or dystrophic mice treated with OPG or anti-RANKL all presented with an improved skeletal muscle function [9, 18, 19]. Also, human intervention studies have reported that in contrast to bisphosphonates, 3 years of denosumab treatment improves the lean mass and handgrip strength of osteoporotic women [20]. This compound has not been tested widely in DMD patients although there are two published case reports of the use of denosumab in an adolescent and adults with DMD [21, 22]. Denosumab holds promise in not only improving skeletal health but potentially also improving muscle outcome; however, its effect has not yet been characterized in a DMD mouse model challenged by GC.

In postmenopausal osteoporosis, characterized by high bone turnover with increased bone resorption, a single dose of intravenous bisphosphonate is clinically recommended to mitigate the rapid bone loss and increased risk of vertebral fractures upon discontinuation of denosumab [23, 24]. This is because unlike bisphosphonates, denosumab is not incorporated into the bone matrix and therefore, the denosumab effect on bone resorption halts after treatment discontinuation [25, 26]. Importantly, there are no pre-clinical or clinical data of this strategy in juvenile animals/growing children or in low bone turnover osteoporosis which is characteristic of patients with DMD, treated with GC.

In this study of dystrophic *mdx* mice, the primary aim was to determine if anti-RANKL treatment improved skeletal muscle function and prevented steroid-induced bone loss. We also aimed to establish whether administration of repeated doses of intravenous bisphosphonate following discontinuation of anti-RANKL in *mdx* mice treated with GCs leads to the stabilization of bone mass. The ability of one drug to prevent bone loss and impact on skeletal muscle outcomes in boys with DMD treated with GC could lead to a step-change in their clinical management.

## Methods

### Animals

All animal experiments were approved by the Université Laval Research Center Animal Care and Use Committee based on The Canadian Council on Animal Care guidelines. Male wild-type (WT) (C57BL/6J) and *mdx* (C57BL/10ScSn-Dmd*mdx*/J) mice were initially purchased from the Jackson Laboratory (Bar Harbor, Maine, ME) and were bred in a specific pathogen-free animal facility. The mice were housed under a 12:12-h light/dark cycle with food ad libitum.

### Study 1: The Effect of Anti-RANKL Treatment on Muscle Pathology and Bone Structure of *mdx* Mice

**Fig. 1:**
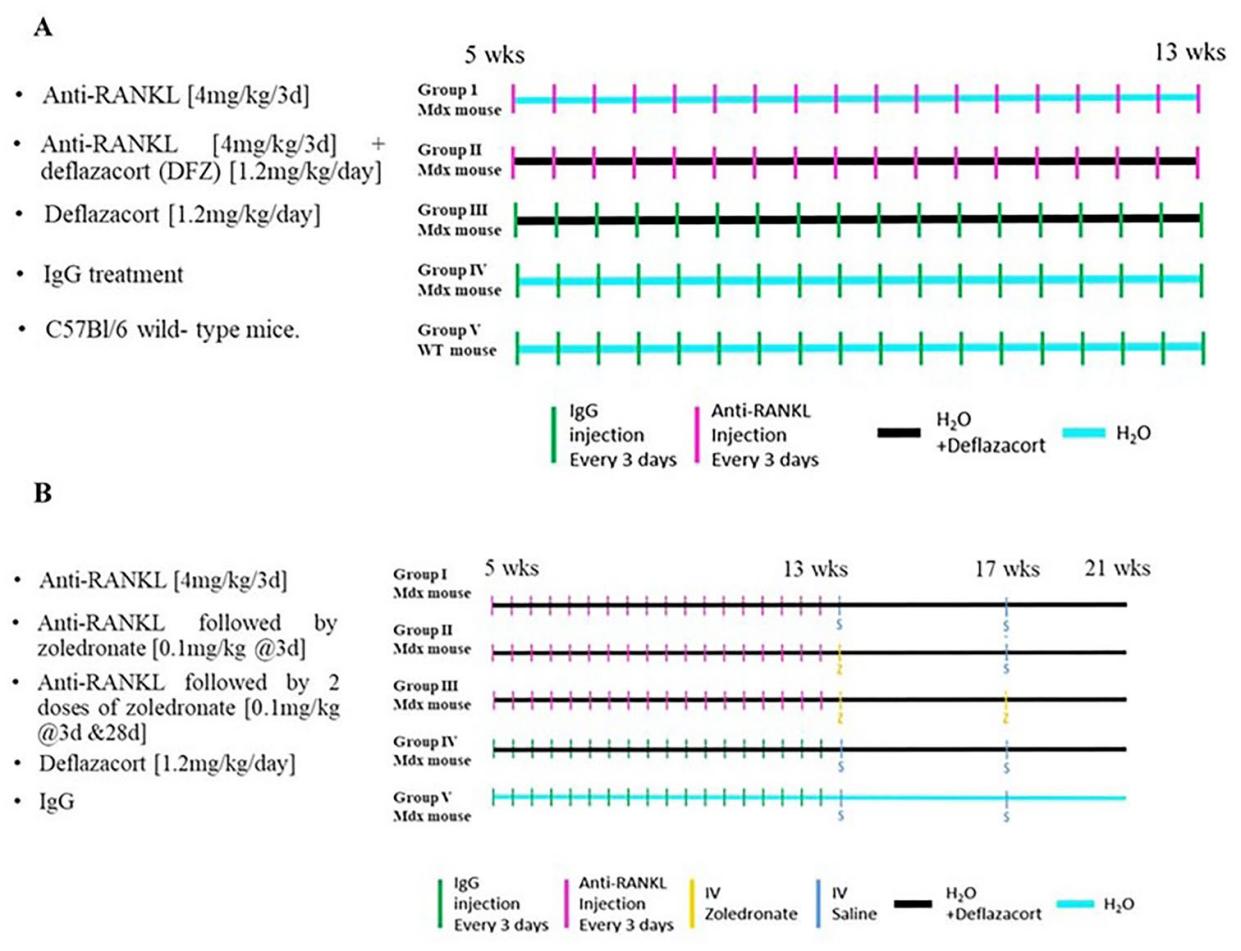
Schematic of experimental design of both in vivo studies. **A** Does anti-RANKL prevent muscle and bone damage in dystrophin-deficient *mdx* mice. **B** Does anti-RANKL treatment alone or followed by bisphosphonate improve bone structure and biomechanical properties in DFZ-treated dystrophin-deficient *mdx* mice

Five-week-old *mdx* mice were divided into 4 treatment groups with each containing 5–8 mice (Fig. 1A).

Group I: anti-mouse RANKL mAbs (IK22-5) [4 mg/kg] every 3 days [27]

Group II: combination of anti-RANKL [4 mg/kg] and DFZ [1.2 mg/kg/day].

Group III: deflazacort (DFZ) [1.2 mg/kg/day] in drinking water with IgG [4 mg/kg] injections.

Group IV: control IgG [4 mg/kg] every 3 days.

A 5th group of WT mice (Group V) received control IgG [4 mg/kg] every 3 days.

Water intake was measured in all experimental groups (mean = 3 mL/day per mouse). The mice were weighed twice weekly to determine the appropriate drug dose and to monitor growth. At the end of the experimental procedures (8 weeks), the mice were administrated buprenorphine for analgesia [i.p. 0.1 mg/kg] and sodium pentobarbital for anesthesia; then, the extensor digitorum longus (EDL) muscles from the left hind limbs were removed to assess their contractile properties and the anesthetized mice were euthanized by cervical dislocation. The EDL muscles from the right hind limbs were also dissected, snap frozen, and stored at − 80 °C for immunofluorescence staining. For skeletal analysis, the left tibia and L6 vertebra were dissected and stored at − 20 °C for micro-computed tomography (μCT) and biomechanical testing.

#### Grip Force Test

The whole limb grip force test was performed on mice before and after 4 or 8 weeks of treatment. Each mouse was held by its tail and grasped horizontally with its four paws placed on the metal grid attached to a digital force meter (Columbus Instruments). The highest force produced during pulling was recorded. The grip force test was repeated three times with at least a 1-min rest between measurements. Maximum grip force was normalized to body mass [28].

#### Assessment of Skeletal Muscle Contractile Properties

After dissection, the EDL muscle was attached to an electrode and a force sensor (305B-LR dual-mode, Aurora Scientific Inc.) controlled by Dynamic Muscle Control Analysis unit and data acquisition software (Aurora Scientific Inc.). The EDL muscle was incubated at 25 °C in oxygenated Krebs–Ringer solution with [2 mg/mL] of added glucose. Once the optimal length (L0) had been determined, the muscle was stimulated for 500 ms at 1, 10, 20, 35, 50, 80, 100, 120, and 150 Hz to induce sub-tetanic and tetanic contractions and to determine the force–frequency curves. Twitch force (Pt, g) and maximal absolute force (*P*_0_, g) values were recorded and analyzed using Dynamic Muscle Data Analysis software (Aurora Scientific Inc.). Maximum specific tetanic tension sP_0_ (N/cm_2_) values were obtained by normalizing the absolute force P_0_ with the cross-sectional area (CSA) using the following equation: sP_0_ = *P*_0_/CSA. CSA was determined by dividing the muscle mass by the product of the optimum fiber length (Lf) corresponding to the result of multiplying L_0_ with the fiber length ratio (0.44 for EDL muscle) and the muscle density (1.06 mg/mm^3^).

#### Immunofluorescence Staining

Transverse EDL muscle cryosections (10 µm) were cut using a refrigerated (− 20 °C) cryostat (Leica Microsystems CM1850). Tissue sections were stained with hematoxylin and eosin to assess muscle damage. Masson’s trichrome staining was used to access collagen infiltration. The damaged area was defined as the area not occupied by normal or regenerating muscle fibers with the presence of infiltrating cells. Digital photographs were acquired from at least five different sections at × 400 magnification and were examined with an inverted microscope (Nikon). Data are expressed as the percentage of damaged and fibrotic areas with respect to the total area using ImageJ (software version 1.41). In other preparations for double-labeling, the sections were washed for 5 min with phosphate buffered saline (PBS), fixed for 10 min with 4% paraformaldehyde (PFA) and then incubated overnight at 4 °C with anti-F4/80 (Bio-Rad, 1:100) and anti-laminin (Sigma-Aldrich, 1:250) antibodies in blocking solution. The sections were washed briefly with PBS, incubated with Alexa Fluor 488 or 594-conjugated secondary antibody (Invitrogen, 1:500) for 1 h at room temperature, and washed three times for 15 min with PBS. The slides were then mounted with Fluoromount-G™, with DAPI immunofluorescent stain and analyzed with an Axio Imager M2 microscope connected to an AxioCam camera using ZEN2 software (Zeiss, Germany).

#### Serum Creatinine Kinase Assay

Blood collected from the mice by cardiac puncture was allowed to clot and was centrifuged at 10,000×*g* for 10 min at 4 °C. The supernatant was transferred to a clean tube for a second round of centrifugation. The serum was then collected and was stored at − 80 °C until used. Serum creatinine kinase (CK) levels, an indicator of muscle damage and sarcolemma membrane fragility in dystrophic mice, were determined using a commercially available kit according to the manufacturer’s instructions (Pointe Scientific Creatinine Kinase CK10 reagent, Fisher Scientific) and a modified protocol from Treat NMD_M.2.2.001 [29]. Serum CK activities were measured using a microplate reader (Infinite F200, TECAN) and were expressed as U/L.

#### Cortical and Trabecular Bone Analysis by Microcomputed Tomography

The changes in trabecular architecture and cortical geometry of both L6 vertebrae and left tibia were assessed by μCT (Skyscan 1172, Bruker, Kontich, Belgium). For trabecular and cortical analysis, high-resolution scans with an isotropic voxel size of 5 µm were acquired (60 kV, 167 μA, 0.5 mm aluminum filter, 0.6° rotation angle). Two images were averaged at each rotation angle. From the reconstructed images obtained using Skyscan NRecon software v1.6.9 (Bruker, Belgium), CTAn software 1.15.4.0 (Skyscan) was used to visualize and determine the bone histomorphometric parameters. For vertebrae, a 300-slice subset through the middle of vertebrae’s body was analyzed. In the proximal tibial metaphysis, a 250-slice subset of trabecular bone extending distally 5% from the base of the growth plate and excluding both the cortical shell and primary spongiosa was analyzed. Cortical bone analysis was performed on a slice subset derived from μCT scan images at 10–90% of total bone (Hsu et al. 2022). To assess the bone mineral density (BMD), BMD phantoms of known calcium hydroxyapatite mineral densities of 0.25 and 0.75 g/cm^3^ were scanned and reconstructed using the same parameters as used for bone samples.

#### Biomechanical Testing

The L6 vertebra was evaluated by compression testing using a LS5 LLOYD testing machine with the NEXYGEN Plus software (AMETEK, UK). From each animal, the vertebral body was isolated from the spinal processes and prepared with flat and parallel ends using a polishing wheel (Dremel, UK). The vertebra was bonded to a fixed bottom plate with cyanoacrylate glue and a top plate (500 N load cell) moved downwards at a speed of 10 mm/min, compressing the vertebra. Each vertebra was tested to fracture and data recorded after every 0.2 N change in load. Yield load, load at maximum stiffness, work to failure, and work to fracture were calculated [30, 31]. The load–displacement curve for each bone was analyzed, and the functional properties of the bone were quantified [31, 32] (Supp. Fig.1).

#### Serum Analysis for Bone Turnover Markers

Serum was analyzed by enzyme-linked immunosorbent assay (ELISA) to measure N-terminal propeptide of procollagen type I (PINP, Wuhan Fine Biotech, China) and α-carboxy-terminal telopeptide of type I collagen (αCTX, Wuhan Fine Biotech, China) levels according to the manufacturer’s instructions.

### Study 2: The Effect of Anti-RANKL Treatment Alone or Followed by Bisphosphonate on the Bone Structure and Mechanical Properties of GC-Treated Dystrophic *mdx* Mice

Five-week-old DFZ [1.2 mg/kg/day in drinking water]-treated *mdx* mice were divided into 4 treatment groups with each containing 5–8 mice (Fig. 1B).

Group I: anti-RANKL [4 mg/kg] every 3 days for 8 weeks followed by two saline injections at 3 and 28 days after cessation of anti-RANKL.

Group II: anti-RANKL [4 mg/kg] every 3 days for 8 weeks followed by a single dose of zoledronate (Zol) [0.1 mg/kg] 3 days after cessation of anti-RANKL and one saline injection 28 days after cessation of anti-RANKL [33].

Group III: anti-RANKL [4 mg/kg] every 3 days for 8 weeks followed by 2 doses of Zol [0.1 mg/kg]) 3 and 28 days after cessation of anti-RANKL.

Group IV: control IgG [4 mg/kg] every 3 days for 8 weeks followed by two saline injections at 3 and 28 days after cessation of anti-RANKL.

A 5th group of *mdx* (without DFZ) mice received control IgG [4 mg/kg] every 3 days for 8 weeks followed by two saline injections at 3 and 28 days after cessation of control IgG.

All mice were killed after 16 weeks of treatment. Water intake was measured in all experimental groups (mean = 3 mL/day per mouse). The mice were weighed twice weekly to determine the appropriate drug dose and to monitor growth. At the end of the experimental procedures (16 weeks), the mice were euthanized by cervical dislocation under anesthesia. The left tibia and L3 vertebrae were dissected and stored at − 20 °C for μCT and biomechanical testing (analyzed as described in Aim 1 for vertebra). For the left tibia, the biomechanical properties were determined by 3-point bending using the Lloyds materials testing machine fitted with a 100N load cell. Tibia were positioned horizontally on custom supports. The load was applied perpendicular to the mid-diaphysis and the cross-head was lowered at 10 mm/min whereas the right tibia and L5 were fixed in 4% PFA for 24 h, decalcified in 10% EDTA for 2 weeks, and embedded in paraffin wax following standard procedures**.**

#### Osteoclast and Osteoblast Quantification

To detect the osteoclasts with tartrate acid phosphatase (TRAP) activity, 70 mg naphthol AS-TR phosphate (Sigma-Aldrich, UK) was dissolved in 250 µL *N*–*N* dimethyl formamide (Sigma-Aldrich, UK) and added to 50 mL of 0.2 M sodium acetate buffer (pH 5.2) containing 2.3 mg/ml sodium l-tartrate dibasic dihydrate (Sigma-Aldrich, UK) and 1.4 mg/mL fast red salt TR (Sigma-Aldrich, UK). Slides were incubated in a water bath kept at 37 ℃ for 60 min. Sections were counterstained in Meyer’s hematoxylin (Sigma, UK), washed in distilled water, and mounted in an aqueous mounting medium (Dako, USA). Slides were imaged using a NanoZoomer-XR slide scanning system (Hamamatsu Photonics, Japan). In the same sections, hematoxylin stained osteoblasts were scored per millimeter trabecular bone surface using morphological criteria in line with previously published studies [34, 35]. Static histomorphometry was quantified using the BIOQUANT OSTEO (BIOQUANT Image Analysis Corporation, Texas, USA) software package using the approved ASBMR histomorphometry nomenclature [36]. Two sections from each mouse were analyzed.

#### Serum Analysis for Bone Turnover Markers

Serum was analyzed by ELISA as described for study 1.

### Statistical Analyses

All values are expressed as means ± SEM. The data were analyzed using Prism (version 3.1) with one-way ANOVA or two-way ANOVA with Turkey’s post hoc test. Details of the specific test used is provided in the legends of each figure and table. The levels of significance were set at *p < 0.05, **p < 0.01, ***p < 0.001, and ****p < 0.0001.

## Results

### Study 1: Both Anti-RANKL and DFZ Treatments Improve Dystrophic Muscle Function But Anti-RANKL + DFZ Co-treatment Did Not Show Any Additive Effect

**Fig. 2:**
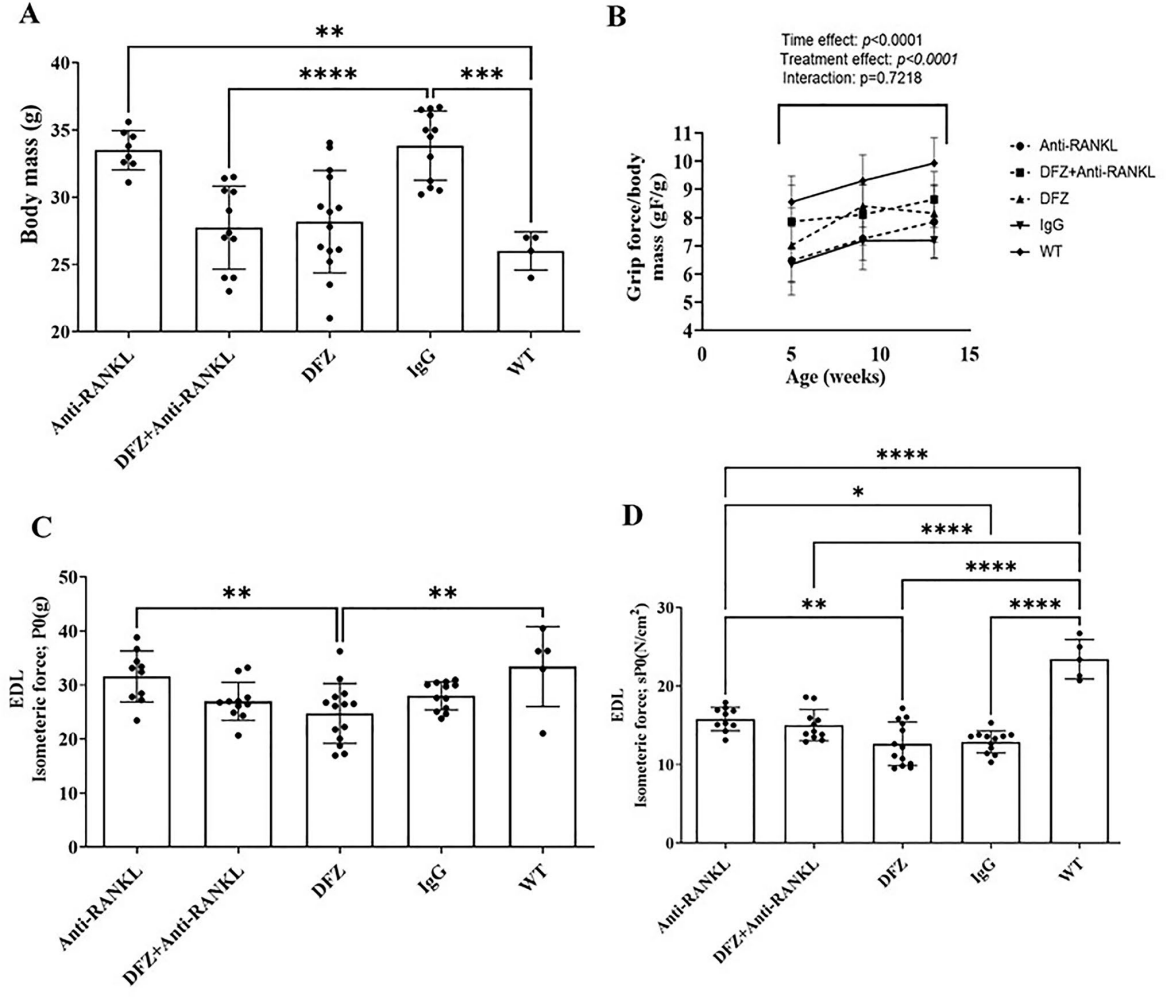
The anti-RANKL and DFZ treatments significantly improved whole limb grip force performance without any synergetic effects of the combined treatment. Glucocorticoid + Anti-RANKL-treated *mdx* and WT mice had significantly lower body mass compared to IgG-treated mice at 8 weeks (**A**). Whole limb grip force was measured before treatment (5 weeks of age) and after 4 and 8 weeks of treatment. Whole limb grip force increased significantly with age in all groups except for the placebo-IgG-treated *mdx* mice (**B**). The anti-RANKL treatments, but not DFZ, significantly improved contractile properties of dystrophic muscles. The contractile properties of the EDL muscle were evaluated ex vivo (**C**). The anti-RANKL treatment significantly improved the specific force (sP0) of dystrophic EDL when compared with IgG-treated *mdx* mice (**D**). Healthy EDL muscles from age-matched WT C57BL6/10 J mice served as controls for contractile property recordings. Data in **A**, **C,** and **D** are expressed as means ± SEM. *p < 0.05, **p < 0.01, ***p < 0.001, and ****p < 0.0001 using one-way ANOVA with the Tukey correction for multiple comparisons. Data in **B** are expressed as means ± SEM. *p < 0.05, **p < 0.01, ***p < 0.001, and ****p < 0.0001 using two-way ANOVA

To assess the long-term effects of anti-RANKL and DFZ on dystrophic skeletal muscle function, 5-week-old *mdx* mice were treated with DFZ or anti-RANKL, or both for 8 weeks (Fig. 1A). Mice treated with anti-RANKL + DFZ and those from the WT group had lower body mass (BM) when compared to the IgG-treated *mdx* mice (Fig. 2A). To evaluate the effect of the treatments on muscle mass, muscles were weighed, and their mass was normalized to body mass. As expected, the EDL muscle of IgG-treated *mdx* mice had higher muscle mass (52.3%) and muscle mass/BM ratios (31.3%; Table 1) when compared to IgG-treated WT mice. Interestingly, 8 weeks of treatment with anti-RANKL, but not DFZ, significantly decreased EDL muscle mass/BM ratio by 9.5% (Table 1). Co-treatment with anti-RANKL + DFZ slightly decreased the EDL muscle mass/BM ratio by 7.1% when compared with the IgG-treated *mdx* EDL (Table 1).

**Table 1:** Muscle mass of EDL muscle

Groups	EDL	
	Muscle mass (mg)	Muscle mass/BM (mg/g)
WT-IgG	9.01 ± 0.5 (****)	0.32 ± 0.02 (****)
*mdx-*IgG	13.72 ± 0.3	0.42 ± 0.01
*mdx-anti-RANKL*	12.68 ± 0.3	0.38 ± 0.01(*)
*mdx-DFZ*	11.68 ± 0.5(**)	0.41 ± 0.01
*mdx-DFZ* + *anti-RANKL*	11.01 ± 0.4(***)	0.39 ± 0.01(*)

Data are expressed as means ± SEM

*BM* body mass

*p < 0.05, **p < 0.01, ***p < 0.001, and ****p < 0.0001 indicate significantly different from the IgG-treated *mdx* mice using one-way ANOVA

The whole limb grip force was measured at 5, 9, and 13 weeks of age and analysis of the data disclosed a significant age-dependent increase of the grip force normalized to body mass (gF/gBM) in all the mice (Fig. 2B). The whole limb grip force of IgG-treated *mdx* mice was significantly lower than that of WT mice. Compared with the IgG-treated *mdx* mice, the grip force was improved by 16% and 13% in DFZ-treated *mdx* mice at 9 and 13 weeks, respectively. Grip force of dystrophic mice treated with anti-RANKL or anti-RANKL + DFZ at 13 weeks was significantly higher compared to the treatment with IgG alone (8.3 ± 0.4, 8.6 ± 0.3, respectively, vs. 7.1 ± 0.2; Fig. 2B). Although significantly different from the IgG-treated *mdx* mice, the anti-RANKL + DFZ combined treatment did not show any additive effects on whole limb grip force (Fig. 2B).

#### Anti-RANKL Treatment, But Not DFZ, Improves the Contractile Properties of Dystrophic Muscles, and With No Additive Effect with Anti-RANKL and DFZ Co-treatment

Anti-RANKL treatment did not improve the absolute tetanic force (P0); however, it resulted in a significant increase in the maximum specific force (sP0) of dystrophic EDL muscles compared to IgG-treated *mdx* mice (Fig. 2C, D). Anti-RANKL increased EDL sP0 by 22.6% in comparison with IgG-treated muscles (Fig. 2D). The combined anti-RANKL + DFZ treatment did not have an additive effect on EDL muscle contractility. DFZ treatment alone did not produce any gain in dystrophic EDL muscle function (Fig. 2D).

#### Anti-RANKL Treatment Alone is as Effective as DFZ and Anti-RANKL + DFZ Co-treatment in Reducing Dystrophic Muscle Damage, Fibrosis, and Neutrophil Infiltration

**Fig. 3:**
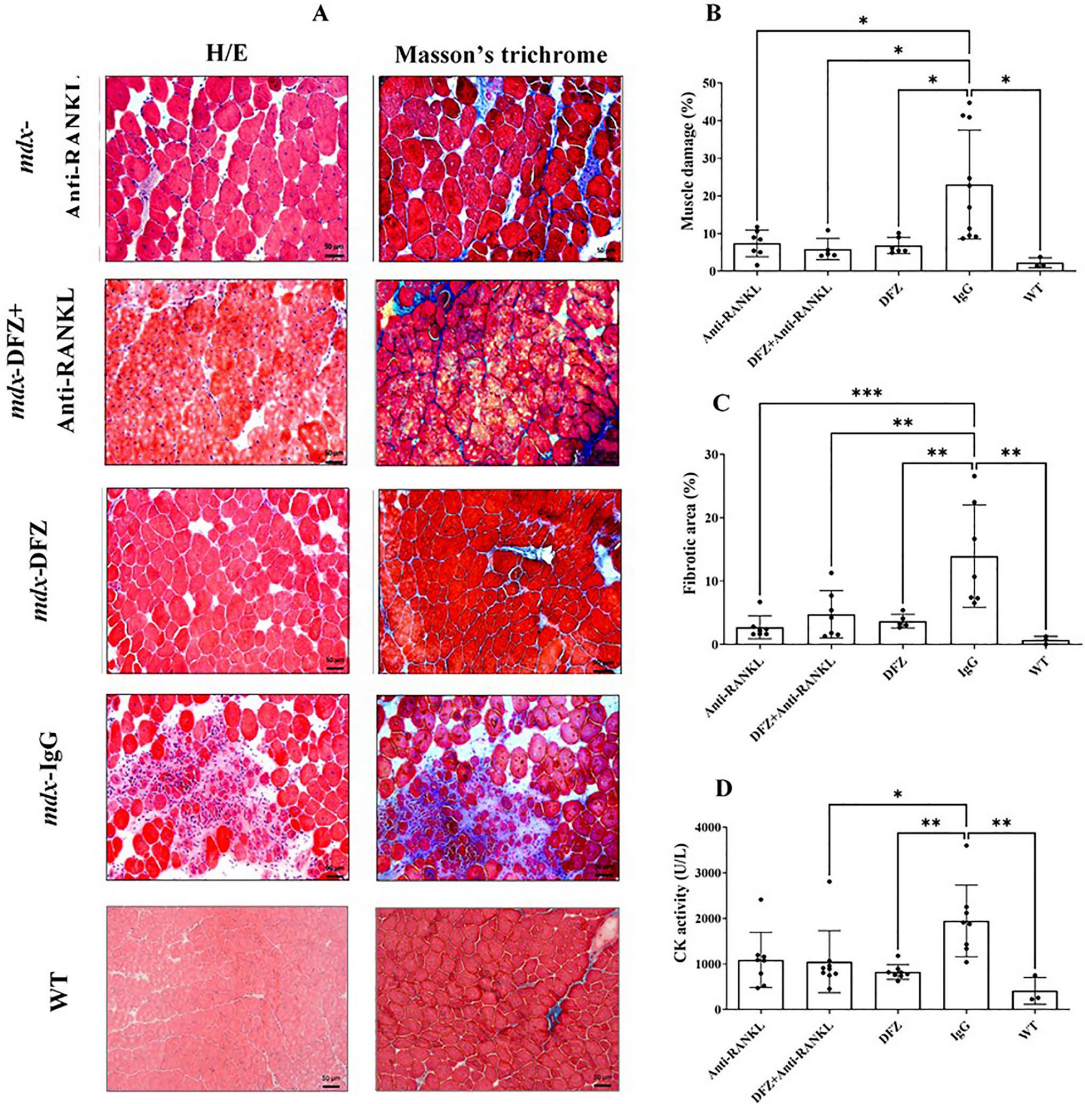
Anti-RANKL and DFZ or anti-RANKL + DFZ co-treatment significantly improved muscle integrity to the same extent but did not have the same effect on muscle regeneration. Representative hematoxylin/eosin and Masson’s trichrome-stained histological sections of EDL muscles from *mdx* mice treated for 8 weeks with either vehicle IgG [4 mg/kg/3 days], anti-RANKL [4 mg/kg/3 days], DFZ [1,2 mg/kg/days] in drinking water or the combined treatment with anti-RANKL and DFZ (**A**). Compared with the IgG-treated *mdx* mice and WT mice, all treatment groups displayed a significant reduction of muscle damage and fibrotic areas (**B** and **C**, respectively) and serum CK activity (**D**). Healthy EDL muscles from age-matched WT C57BL6/10J mice served as controls. Muscle damage and fibrosis were quantified using ImageJ software excluding the edges of the sections. Data are expressed as means ± SEM. Significantly different from IgG-treated *mdx* mice, *p < 0.05 and **p < 0.01, ***p < 0.001, and ****p < 0.0001 using one-way ANOVA with the Tukey correction for multiple comparisons. Scale bar = 50 µm

**Fig. 4:**
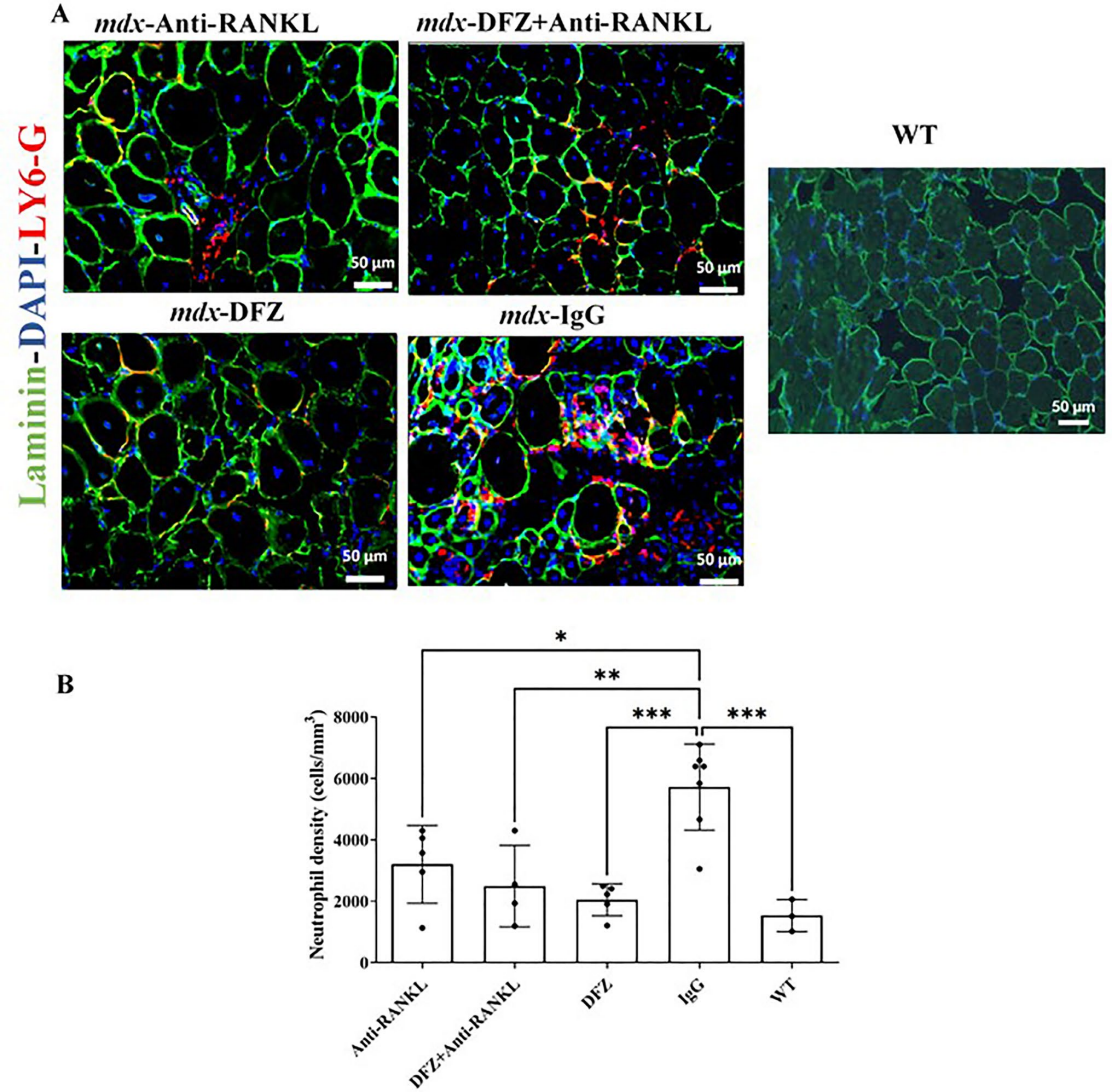
Anti-RANKL and DFZ treatments significantly reduced neutrophil cell infiltration without any additive effect with the combined treatments. Cryosections of EDL muscles from *mdx* mice treated for 8 weeks with either vehicle IgG [4 mg/kg/3 days], of anti-RANKL [4 mg/kg/3 days], DFZ [1.2 mg/kg/days] in drinking water or the combined treatment with anti-RANKL and DFZ were labeled with anti-Ly6-G/C (to label neutrophils; red), anti-laminin (green), and DAPI (blue) (**A**). Compared with the IgG-treated *mdx* mice and WT mice, all treatments groups had significantly reduced number of neutrophils (**B**). Muscles from WT C57BL/10J mice served as controls. Data are expressed as means ± SEM. **p < 0.01, ***p < 0.001 indicate significantly different from the IgG-treated mice using one-way ANOVA with the Tukey correction for multiple comparisons. Scale bar = 50 µm (Color figure online)

We next investigated the effects of anti-RANKL treatment and/or DFZ on muscle damage and fibrosis, one of the most distinctive features of dystrophic muscles. As expected, muscle damage was significantly elevated in IgG-treated *mdx* mice compared to WT mice (Fig. 3A). Analysis of H&E stained sections revealed a marked reduction in the damaged and fibrotic area of dystrophic EDL muscles from the three experimental treatments (5.9–7.4%) when compared with IgG-treated *mdx* mice (23.1%; Fig. 3B, C). The fibrotic area, as assessed by Masson trichrome staining, in the dystrophic EDL was significantly lower in the three experimental treatment groups compared with IgG-treated *mdx* mice (2.7–4.8% vs. 14.0%, respectively; Fig. 3A, C). In terms of muscle damage and fibrosis, the combined treatment of anti-RANKL and DFZ was not superior to anti-RANKL alone (Fig. 3B, C). Serum CK levels, an indirect indicator of muscle damage, were as expected, higher in IgG-treated *mdx* mice compared with WT mice (Fig. 3D). DFZ, anti-RANKL (NS), and anti-RANKL + DFZ co-treatments reduced serum CK levels by 58%, 44%, and 46%, respectively, compared with the IgG-treated *mdx* mice (Fig. 3D). To investigate the possibility that the decrease in muscle damage is correlated with low inflammatory cell infiltration, EDL muscle sections were labeled with anti-Ly6-G/C, a marker for neutrophils (Fig. 4A). The anti-RANKL, DFZ, and anti-RANKL + DFZ co-treatment significantly reduced the number of neutrophils in the EDL muscle compared to IgG-treated *mdx* mice (3202 ± 566, 2050 ± 233, 2495 ± 663, respectively, vs. 5719 ± 530 cells/mm^3^, Fig. 4B).

#### Anti-RANKL Treatment Improved Bone Structure and Biomechanical Properties of Dystrophic Mice

**Fig. 5:**
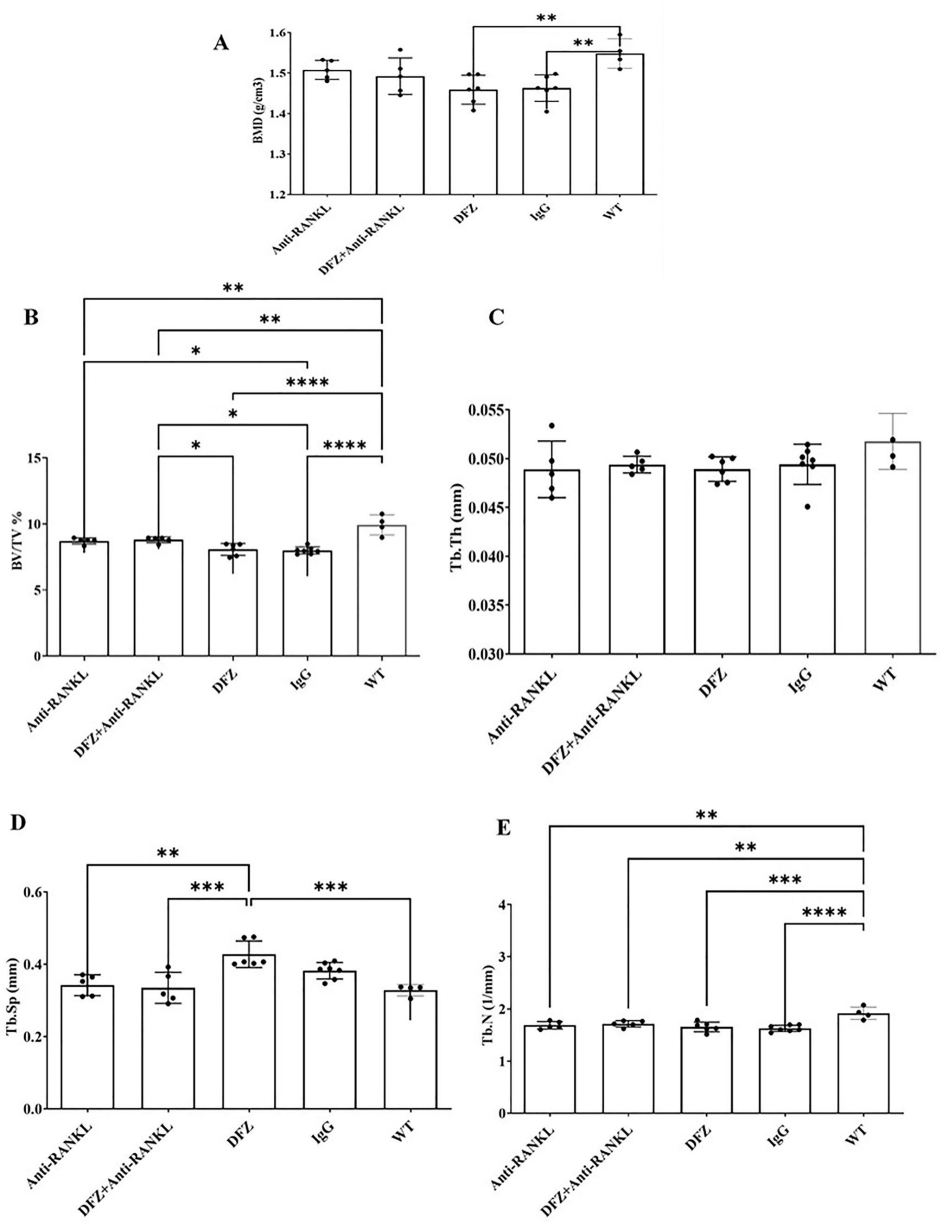
Vertebrae cortical BMD was not significantly decreased in anti-RANKL or anti-RANKL + DFZ-treated *mdx* mice compared to WT mice whereas it was significantly decreased in DFZ and IgG-treated *mdx* mice (**A**). BV/TV (trabecular bone volume/tissue volume; %) was significantly increased in anti-RANKL + DFZ-treated *mdx* mice compared to DFZ-treated *mdx* mice (**B**). Tb.Sp. (trabecular separation; mm) was decreased in anti-RANKL and anti-RANKL + DFZ-treated *mdx* mice compared to DFZ-treated *mdx* mice (**D**). Tb. N. (trabecular number; mm^−1^) and Tb. Th. (trabecular thickness; mm) were not significantly changed in anti-RANKL or anti-RANKL + DFZ-treated *mdx* mice compared to IgG-treated *mdx* mice (**C**, **E**). These data are represented as the means ± SEM. *p < 0.05; **p < 0.01; ***p < 0.001, ****p < 0.0001 using one-way ANOVA with the Tukey correction for multiple comparisons

**Fig. 6:**
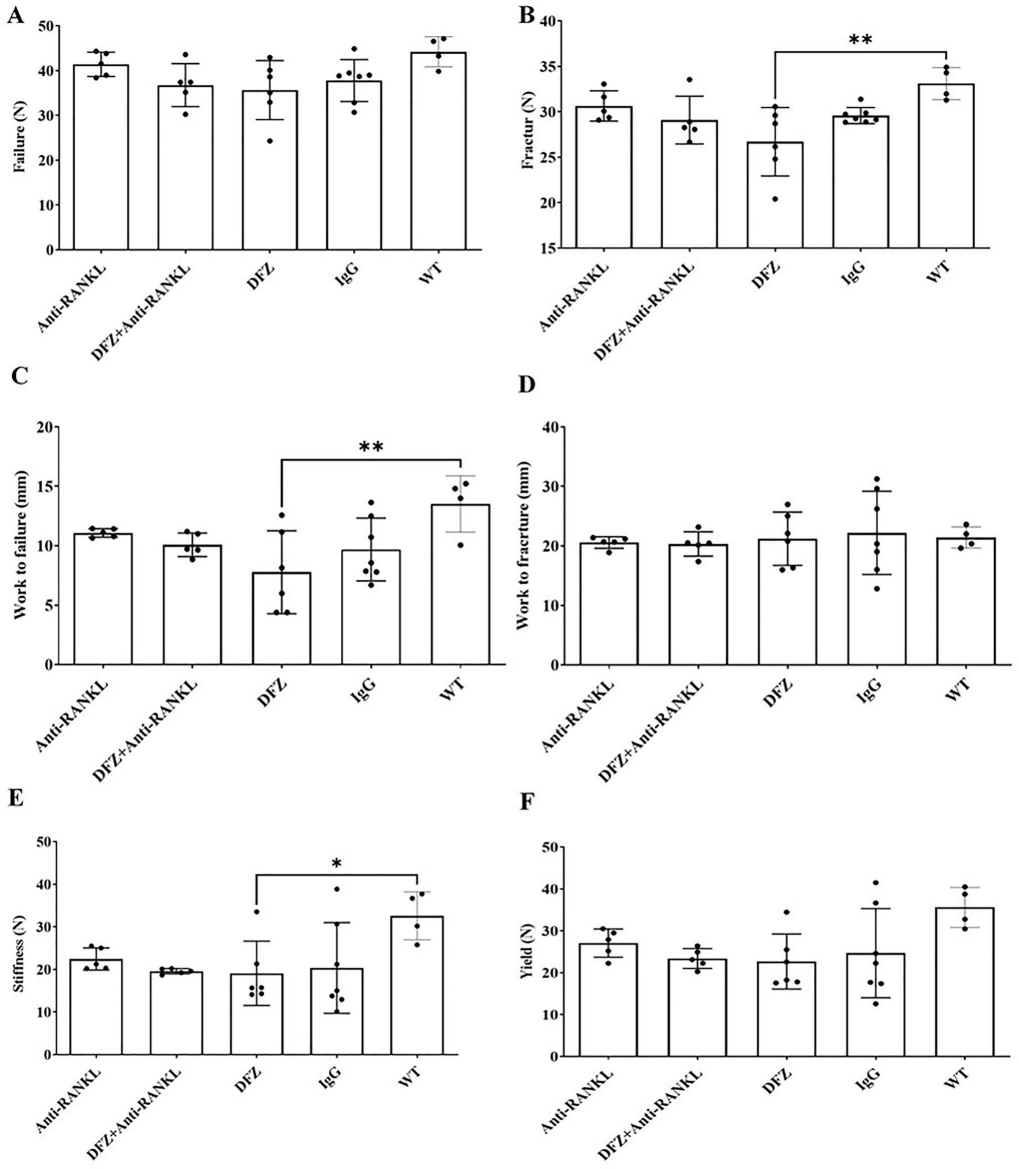
Fracture load (**B**), work to failure (**C**) and stiffness (**E**) were significantly decreased in vertebrae of DFZ-treated *mdx* mice compared to IgG-treated WT mice. Failure load (**A**), work to fracture (**D**) and yield (**F**) were similar in all groups. Data are expressed as the means ± SEM. *p < 0.05; **p < 0.01 using one-way ANOVA with the Tukey correction for multiple comparisons

The μCT analysis of vertebra bone structure revealed that treatment of *mdx* mice with DFZ for 8 weeks had little effect on cortical BMD and various trabecular structural parameters when compared to IgG-treated *mdx* mice. However, in comparison with WT mice, DFZ and IgG-treated mdx mice had lower cortical BMD and trabecular BV/TV, Tb.N, and higher Tb.Sp (Fig. 5). Anti-RANKL treatment with or without DFZ had similar effects on all parameters studies and notably it resulted in higher Tb BV/TV and lower Tb.Sp in *mdx* mice when compared to *mdx* mice treated with DFZ only (Fig. 5). Similar trends were also observed with the tibia (Supp. Figure 2). Compression testing of the vertebra revealed that all the parameters measured were similar in all treatment groups. Exceptions to this included fracture, work to failure, and stiffness which were lower in the DFZ-treated *mdx* compared with WT mice (Fig. 6).

#### Anti-RANKL Treatment Did Not Alter Bone Turnover Over Serum Markers

In comparison with DFZ-treated *mdx* mice, there was a tendency for bone resorption (CTX) and formation (P1NP) serum markers to be decreased and increased, respectively, in anti-RANKL-treated *mdx* mice but these differences did not reach significance (Table 2).

**Table 2:** Serum concentration of bone turnover markers (CTX and P1NP) after treatment of *mdx* mice with IgG, anti-RANKL, DFZ, or anti-RANKL + DFZ

	*mdx*-anti-RANKL	*mdx*-DFZ + anti-RANKL	*mdx*-DFZ	*mdx*-IgG	WT-IgG
CTX (ng/ml)	2.8 + 2.0	3.2 + 1.6	4.3 + 3.0	3.7 + 0.5	2.2 + 0.2
P1NP (ng/ml)	51.5 + 15.8	50.0 + 25.2	38.3 + 20.1	42.6 + 22.8	36.5 + 3.6

Data are expressed as the means ± SEM and analyzed using one-way ANOVA

### Study 2: Anti-RANKL Treatment Alone or Followed by Bisphosphonate Improves Bone Structure and Biomechanical Properties of DFZ-Treated Dystrophic *mdx* Mice

**Fig. 7:**
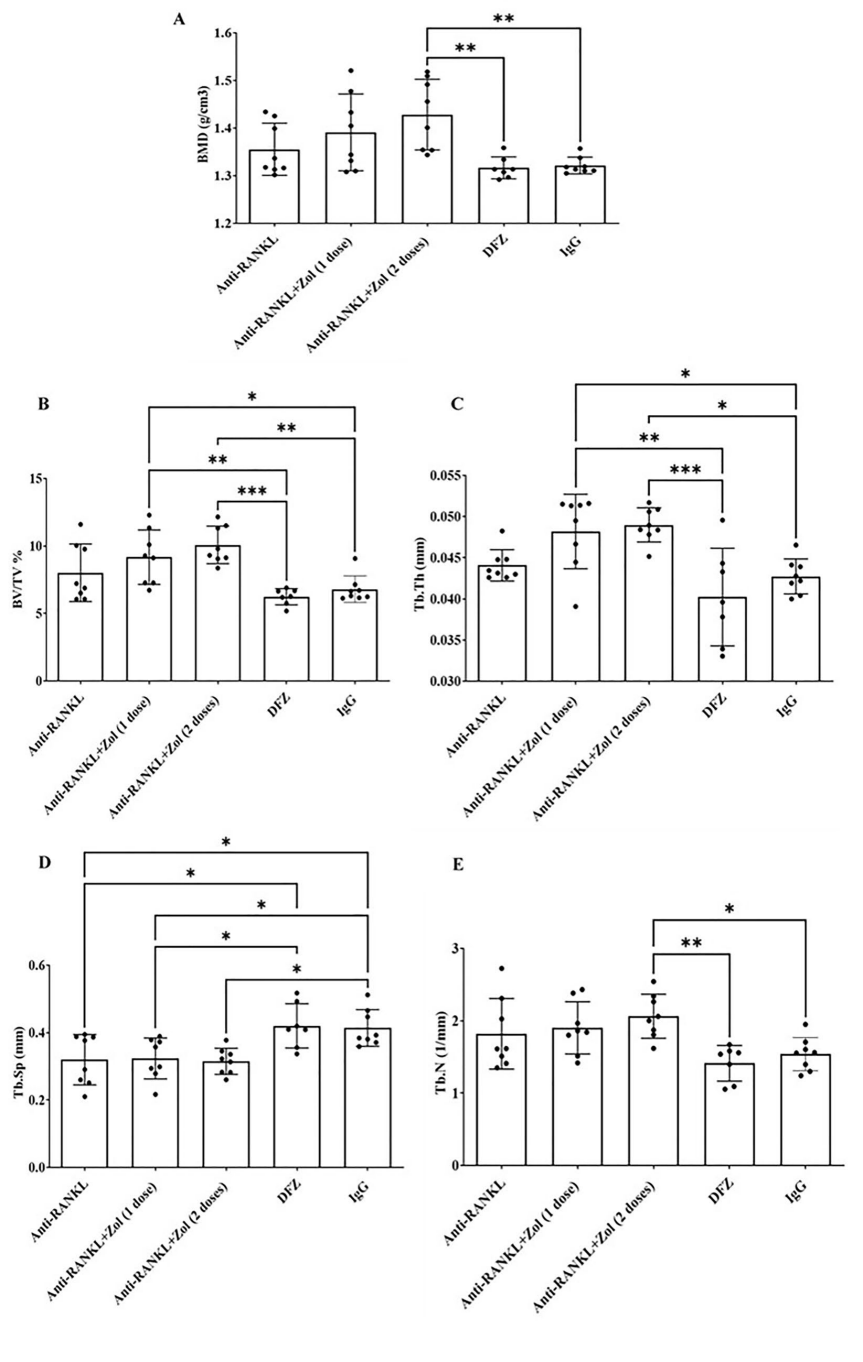
BMD (bone mineral density) and Tb. N. (trabecular number; mm^−1^) were significantly increased in anti-RANKL followed by 2 doses of Zol-treated *mdx* mice compared to DFZ or IgG-treated *mdx* mice (**A**, **E**). BV/TV (trabecular bone volume/tissue volume; %) and Tb. Th. (trabecular thickness; mm) were significantly increased in anti-RANKL followed by 1 or 2 doses of Zol-treated *mdx* mice compared to DFZ or IgG-treated *mdx* mice (**B**, **C**). Tb. Sp. (trabecular separation; mm) was significantly decreased in anti-RANKL, or anti-RANKL followed by 1 or 2 doses of Zol-treated *mdx* mice compared to DFZ-treated *mdx* mice (**D**). Data are represented as the means ± SEM. *p < 0.05; **p < 0.01; ***p < 0.001 using one-way ANOVA with the Tukey correction for multiple comparisons

This study determined whether bisphosphonate administration after anti-RANKL discontinuation was required to prevent GC-induced bone loss and associated biomechanical complications. To do this, we analyzed the bone structure of the vertebra and tibia after 16 weeks of treatment (Fig. 7, Supp. Fig. ). Of the other vertebra parameters measured, only Tb. Sp was altered significantly with anti-RANKL alone when compared to DFZ-treated *mdx* mice. When compared DFZ-treated *mdx* mice, cortical BMD and Tb BV/TV, Tb. Th, and Tb. N all required 1 or 2 doses of Zol after the discontinuation of anti-RANKL to be significantly higher (Fig. 7). Similar to the vertebra DMD, cortical BMD of the tibia was increased in *mdx* mice treated with anti-RANKL followed by 2 doses of Zol compared to DFZ-treated *mdx* mice. Both Tb BV/TV and Tb. Sp were similar in *mdx* mice treated with anti-RANKL or anti-RANKL followed by 1 or 2 doses of Zol and all three treatments significantly increased BV/TV and decreased Tb. Sp when compared with DFZ-treated *mdx* mice. Similarly, in comparison with DFZ-treated *mdx* mice, Tb. N was significantly increased in mice treated with anti-RANKL but only when followed by 1 or 2 doses of Zol. There were no treatment effects on Tb. Th (Supp. Fig. 3).

**Fig. 8:**
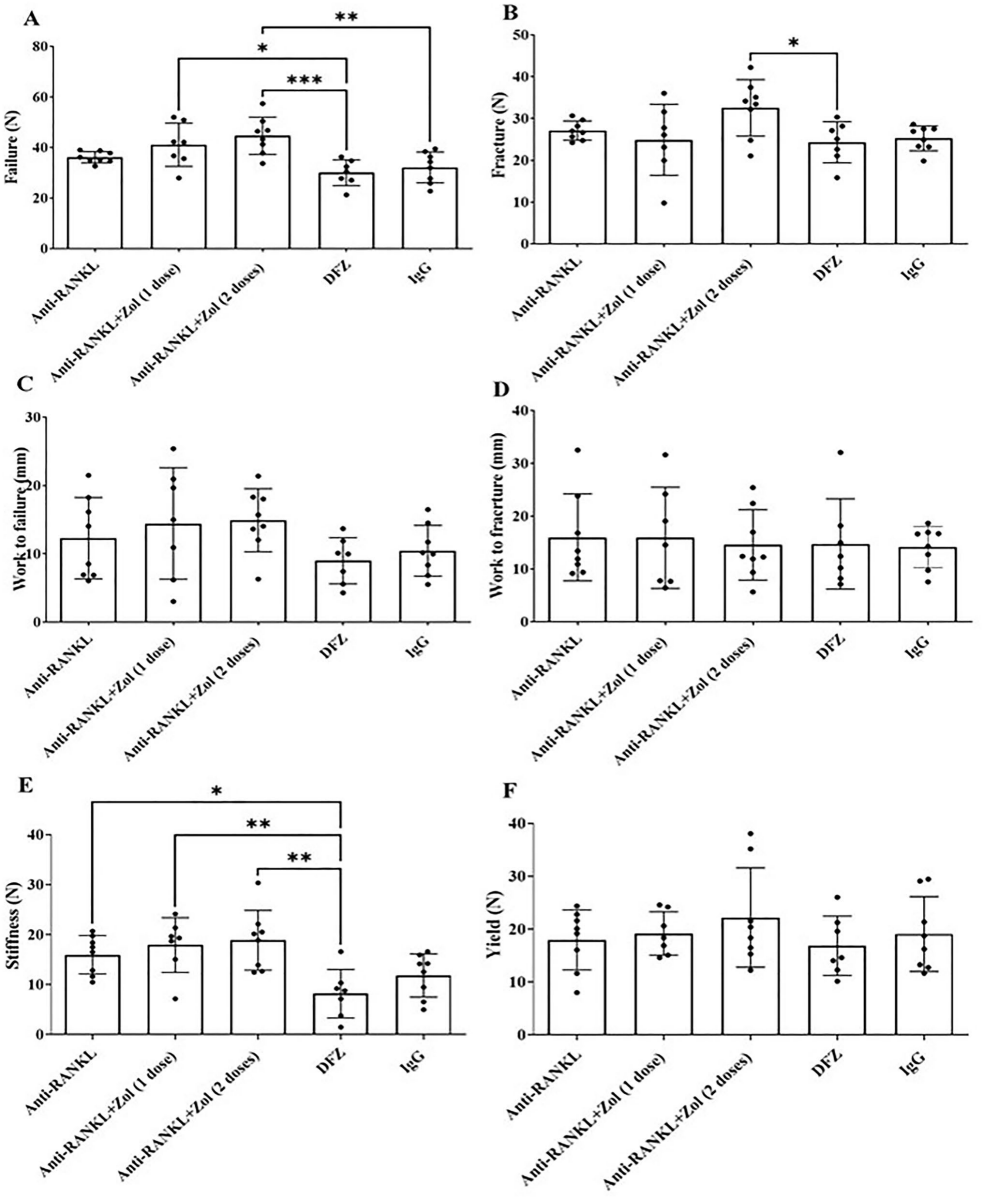
Failure load of vertebrae was significantly increased in anti-RANKL followed by 1 or 2 doses of Zol-treated *mdx* mice compared to DFZ-treated *mdx* mice (**A**). Load at maximum stiffness was significantly higher in anti-RANKL or anti-RANKL followed by 2 doses of Zol-treated *mdx* mice compared to DFZ-treated *mdx* mice (**E**). Fracture load was significantly increased in anti-RANKL followed by 2 doses of Zol-treated *mdx* mice compared to DFZ-treated mdx mice (**B**). Work to failure (**C**), work to fracture (**D**) and yield (**F**) were similar in all groups. Data are represented as the means ± SEM. *p < 0.05; **p < 0.01; ***p < 0.001 using one-way ANOVA with the Tukey correction for multiple comparisons

The biomechanical properties of the vertebra and tibia were determined by compression testing and three-point bending, respectively (Fig. 8, Supp. Fig. 4). In vertebra, anti-RANKL or anti-RANKL followed by 1 or 2 doses of Zol significantly increased load at maximum stiffness compared to DFZ-treated *mdx* mice but the higher failure and fracture loads were absent in mice whose anti-RANKL treatment was discontinued for 8 weeks and not followed by Zol. However, work to failure, work to fracture, and yield strength were similar in all groups (Fig. 8). In tibia, anti-RANKL or anti-RANKL followed by 1 or 2 doses of Zol treatment significantly increased fracture load of the tibia when compared to DFZ-treated *mdx* mice. In contrast, work to failure, load at maximum stiffness, and yield strength of the tibia were significantly greater in DFZ-treated *mdx* mice when 1 or 2 doses of Zol were administered after the discontinuation of anti-RANKL. Work to fracture and failure load were similar in all groups (Supp. Fig. 4).

#### Anti-RANKL Treatment Alone or Followed by Bisphosphonate Decreases Osteoclast Number of DFZ-Treated Dystrophic *mdx* Mice

**Fig. 9:**
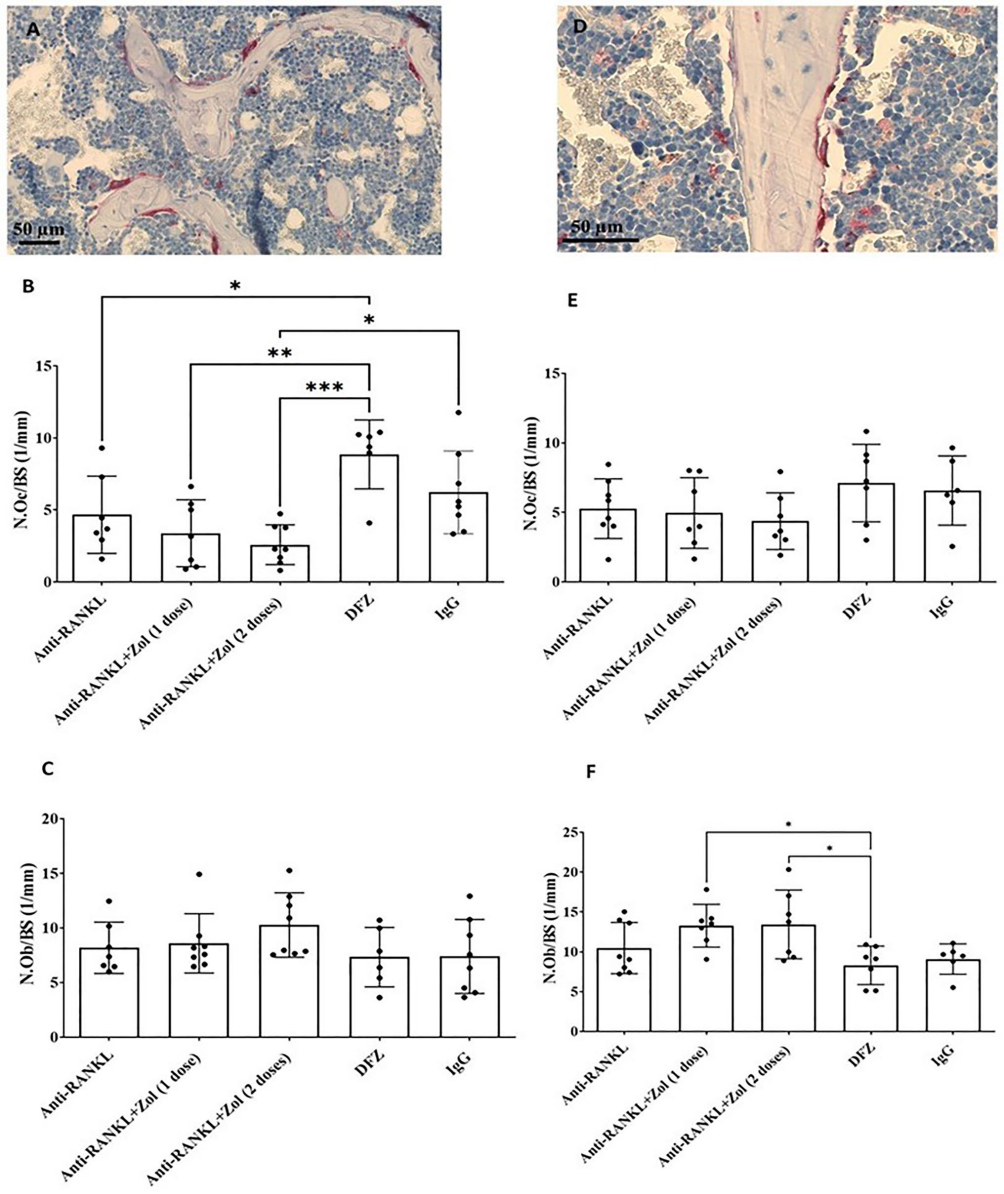
Representative photomicrographs of tibia (**A**) and vertebrae (**D**) sections reacted for tartrate resistant acid phosphatase (TRAP) activity. Anti-RANKL and anti-RANKL followed by 1 or 2 doses of Zol-treated *mdx* mice compared to DFZ-treated *mdx* mice significantly decreased the number of osteoclasts/bone surface (N.Oc/BS) in the tibia (**B**). The number of osteoblasts/bone surface (N.Ob/BS) within the tibia were unchanged by all treatments compared with DFZ-treated *mdx* mice (**C**). The N.Oc/BS in vertebra was unchanged by all treatments compared with DFZ-treated *mdx* mice (**E**). Anti-RANKL followed by 1 or 2 doses of Zol-treated *mdx* mice compared to DFZ-treated *mdx* mice increased N.Ob/BS in vertebra (**F**). Data are expressed as the means ± SEM. *p < 0.05; **p < 0.01; ***p < 0.001 using one-way ANOVA with the Tukey correction for multiple comparisons

The number of osteoclasts/bone surface (N.Oc/BS) in the tibia was significantly reduced in anti-RANKL or anti-RANKL followed by 1 or 2 doses of Zol treatments compared to DFZ-treated *mdx* mice (Fig. 9A, B). No treatment changes were noted in N.Oc/BS in the vertebra (Fig. 9D, E). The number of osteoblasts/bone surface (N.Ob/BS) in the tibia was similar in all groups of mice but was increased in vertebra by anti-RANKL treatment followed by 1 or 2 doses of Zol when compared to DFZ-treated *mdx* mice (Fig. 9C, F).

#### Anti-RANKL Treatment Followed by 1 or 2 Doses of Bisphosphonate Reduces Serum αCTX Concentrations When Compared to DFZ Treatment

In comparison with DFZ-treated mdx mice, the concentration of αCTX was significantly reduced by anti-RANKL alone or followed by 1 or 2 doses of Zol. Higher serum concentrations of P1NP were noted in mice treated with anti-RANKL followed by 2 doses Zol (Table 3).

**Table 3:** Serum concentration of bone turnover markers (CTX and P1NP) after treatment of DFZ-treated *mdx* mice with anti-RANKL, anti-RANKL followed by 1 or 2 doses of bisphosphonate, DFZ, and IgG

	*mdx*-anti-RANKL	*mdx*-anti-RANKL + Zol (1 dose)	*mdx*-anti-RANKL + Zol (2 doses)	*mdx* -DFZ	*mdx*-IgG
CTX (ng/mL)	4.4 + 0.2*	3.1 + 1.6*	3.3 + 1.3*	5.9 + 0.5	5.5 + 1.9
P1NP (ng/mL)	86.2 + 54.7	87.6 + 55.5	90.6 + 45.4*	33.7 + 10.9	44.7 + 11.4

Data are expressed as the means ± SEM

*p < 0.05 indicate significantly different from *mdx-*DFZ mice using one-way ANOVA

## Discussion

The RANK/RANKL/OPG pathway is essential for both bone (re)modeling and any disruption results in bone dysfunction and pathological conditions such osteoporosis [17]. Denosumab is a human monoclonal antibody that neutralizes the activity of human RANKL and in placebo controlled trials, it reduced the incidence of vertebral fractures, non-vertebral fractures, and hip fractures by 68%, 20%, and 40%, respectively [37]. However, the expression of RANK, RANKL, and OPG transcripts and protein are not specific to bone and all three are also expressed in skeletal muscle [38–41]. Similar to bone, changes to the OPG/RANKL ratios in muscle are correlated with dysfunction. Specifically, anti-RANKL administration improves lean body mass and grip strength in osteoporotic women whereas mice overexpressing RANKL exhibit muscle atrophy and weakness [20, 42]. The ability of anti-RANKL therapy to restore muscle function has profound implications for DMD patients because its use offers the possibility of using one drug to improve skeletal muscle function and prevent steroid/muscle function-induced bone loss. The present study investigated the potential for anti-RANKL treatment to prevent GC-induced bone loss and promote muscle function in dystrophic mice. We also examined whether bisphosphonate administration after anti-RANKL discontinuation is required to inhibit a rebound acceleration of bone turnover, despite the low bone turnover state in GIO.

Our results revealed that during an 8-week treatment period, anti-RANKL was as effective as DFZ and the co-treatment with anti-RANKL + DFZ at improving the whole limb grip force of *mdx* mice. When tested ex vivo and consistent with previous results, the anti-RANKL treatment improved the maximum specific force (sP_0_) of the isolated EDL muscle [9]. However, we found DFZ treatment alone did not improve the contractile properties of dystrophic muscles. Previous research by others has revealed that in mice daily GC treatment improved muscle integrity but not muscle strength and function, and grip force while triggering muscle atrophy [43, 44]. The present findings showed no additive effect between anti-RANKL and DFZ treatments on muscle function ex vivo. Indeed, anti-RANKL + DFZ co-treatment increased the force production of dystrophic EDL muscle to the same extent as anti-RANKL alone. These results are consistent with the reduction of the normalized EDL muscle mass/body mass following anti-RANKL treatment and to a lesser extent with anti-RANKL + DFZ co-treatment, suggesting that the gain in specific force is most likely due to a reduction of non-contractile tissues, i.e., edema, fibrosis, or fat cells. Nonetheless, how anti-RANKL and DFZ interact in dystrophic skeletal muscles is very poorly understood and will require further in-depth investigations.

In addition to muscle weakness, DMD patients and *mdx* mice muscles present with an accumulation of muscle inflammation, damage, fibrosis, and high CK activity in the circulation [45, 46]. Here, we showed that the improvements of muscle function with the anti-RANKL and anti-RANKL + DFZ co-treatment were associated with a significant reduction in muscle damage, serum CK levels, and muscle fibrosis suggesting that the integrity of the dystrophic muscles had improved. Muscle integrity was improved following DFZ treatment alone as previously shown on DMD patients and dystrophic mice under GC treatment [43, 47]. Our findings indicated that anti-RANKL, DFZ, and anti-RANKL + DFZ co-treatment significantly reduced the density of neutrophils. Chronic inflammation and leucocyte recruitment are a prominent feature in DMD and the specific depletion of inflammatory cells reduces muscular necrosis and inflammation in *mdx* mice [48, 49]. Our results are of the utmost importance since neutrophils are the first cells to invade damaged muscles [50] and the depletion of host neutrophils resulted in a delay and significant reduction of necrotic myofiber at the acute onset of dystropathology in *mdx* mice [51]. Similarly, we previously found that anti-RANKL treatment alone reduces muscle inflammation and muscle damage thus improving muscle function of dystrophic mice [9]. Moreover, GCs are a well-known anti-inflammatory drug and our results confirm that daily DFZ treatment has a potent effect on muscle inflammation [51]. Overall, anti-RANKL and DFZ treatments may potentially protect the integrity of myofibres by reducing the number of recruited inflammatory cells. While additional muscle benefits of anti-RANKL treatment are unlikely in GC-treated people with DMD, the use of anti-RANKL alone to improve muscle integrity would avoid the use GC and their related skeletal side effects. This would be worthy of investigation in future clinical trials.

Since bone loss occurs with deterioration of muscle function, anti-resorptive therapy may also be an useful approach to inhibit the negative effects of the underlying myopathy and GC therapy on bone [52]. In DMD, the first and most frequent osteoporotic fractures occur in vertebrae after only 6 months of GC therapy, although long bone fracture of lower limb like tibia and femur are extremely common [6, 14]. Therefore, examination of both vertebrae and tibia, as done in this study, provides a comprehensive understanding of the bone response to anti-RANKL treatment. While intravenous bisphosphonates are recommended following fractures to prevent bone loss in GC-treated DMD boys [14], there are significant side effects which includes nausea, vomiting, and pyrexia particularly following first infusion but could also occur in subsequent infusions [53]. Serious complications like rhabdomyolysis and intra-cardiac thrombosis have been reported in people with DMD treated with intravenous bisphosphonates [54–56]. In pre-clinical studies, RANKL inhibition by a human monoclonal anti-RANKL IgG2 antibody prevented GC-induced loss of bone mass and strength in hRANKL-knockin mice [57]. Furthermore, two case studies have also reported improvements in BMD and bone turnover markers in adolescent and adult DMD patients treated with GCs [21, 22]. In this present study, we found that anti-RANKL treatment for 8 weeks improved trabecular bone structure of *mdx* mice which was also noted in *mdx* mice that received both DFZ and anti-RANKL. These results from this and other studies (pre-clinical and clinical) strongly support the benefits of anti-RANKL therapy for the treatment of DMD patients treated with GCs.

A potential concern about the use of denosumab is that unlike bisphosphonates, it is not incorporated into bone. The beneficial effects of denosumab on BMD and bone turnover markers in postmenopausal women with osteoporosis (a high bone turnover state) are reversible upon discontinuation due to an increase in osteoclast number and activity [58–60]. International guidance for postmenopausal osteoporosis suggests that at least a single dose of intravenous bisphosphonates is required to consolidate the gains from the use of denosumab by inhibiting rebound acceleration of bone turnover [23]. Indeed, a number of studies have reported that implementing bisphosphonates after denosumab discontinuation mitigated BMD loss, although the optimal regimen is yet to be clarified [23, 61–63]. However, there is no pre-clinical or clinical data on bone health when denosumab treatment is stopped in animals/growing children or in low bone turnover osteoporosis, e.g., GIO, which is a characteristic of patients with DMD treated with GC. Therefore, the experimental design of the 2nd study incorporated groups of mice that received Zol after the discontinuation of anti-RANKL. We found that one or two doses of Zol after anti-RANKL cessation treatment further enhanced bone microarchitecture parameters and biomechanical properties compared to DFZ-treated *mdx* mice. The biomechanical properties were closely related to the microarchitecture parameters of bone, supporting previous conclusions that microarchitecture could be used to predict the biomechanical properties of trabecular bone [64]. Decreased bone resorption underpins the superior structural and biomechanical properties of bones from anti-RANKL and Zol-treated mice. This was confirmed by the lower serum CTX in mice who received 1 or 2 doses of Zol after the discontinuation of anti-RANKL. Osteoclast number within the tibia were also lower in mice treated with anti-RANKL alone or followed by Zol. Similar results were reported by Hofbauer and colleagues who indicated that denosumab treatment reduced serum TRAP-5b concentrations and distal femur and lumbar vertebra osteoclast number in prednisolone-treated mice [57]. It is therefore sensible that treatment with bisphosphonates is required to limit reactivation of bone turnover after anti-RANKL discontinuation in DMD despite the low bone turnover. Further studies are required to determine if fracture risk is decreased with anti-RANKL treatment alone or with bisphosphonates following anti-RANKL discontinuation in DMD and other states of GC-induced osteoporosis.

In conclusion, anti-RANKL treatment improved bone structure and muscle function in GC-treated *mdx* mice. A clinical trial examining the efficacy on skeletal muscle outcomes and safety of denosumab in comparison with GC in DMD would be worth pursuing. As observed in osteoporotic adult patients, bisphosphonate administration after anti-RANKL discontinuation may be required for patients with DMD to inhibit a rebound acceleration of bone turnover and reduce fracture risk, despite the low bone turnover state in GIO.

### Supplementary Information

Below is the link to the electronic supplementary material.

Supplementary file1 (DOCX 1801 KB)

## Data Availability

All data supporting the findings of this study are available within this article.
