# Supplementary material for: Anti-RANKL Therapy Prevents Glucocorticoid-Induced Bone Loss and Promotes Muscle Function in a Mouse Model of Duchenne Muscular Dystrophy
Source: Calcif Tissue Int. 2023 Jul 20;113(4):449–68. doi: 10.1007/s00223-023-01116-w (PMC10516841; doi:10.1007/s00223-023-01116-w)
Supplement: Supplementary file 1 — Supplementary file1 (DOCX 1801 KB) [file 223_2023_1116_MOESM1_ESM.docx]

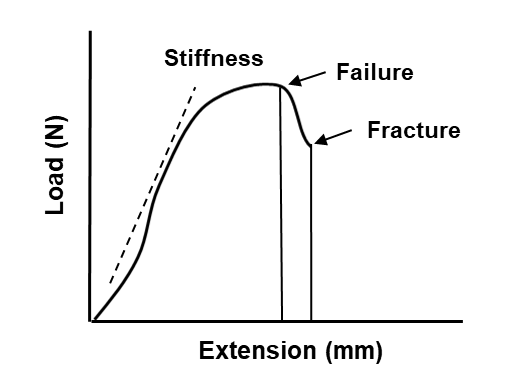


**Supp. Figure 1.** Load–displacement curves illustrating biomechanical parameters including failure, fracture and stiffness. Work to failure is calculated as the area under the curve up to the failure point. Work to fracture is calculated as the area under the curve up to the fracture point.

**Supp. Figure 2.** Treatment of *mdx* mice with anti-RANKL alone or followed by 1 or 2 doses of Zol had no effect on tibia BMD or microstructure when compared with DFZ treated *mdx* mice. Tb. Th. (trabecular thickness; mm) was significantly decreased in all mdx mice (irrespective of treatment) when compared with IgG treated WT mice. Data are expressed as means ± SEM. ****p < 0.0001.


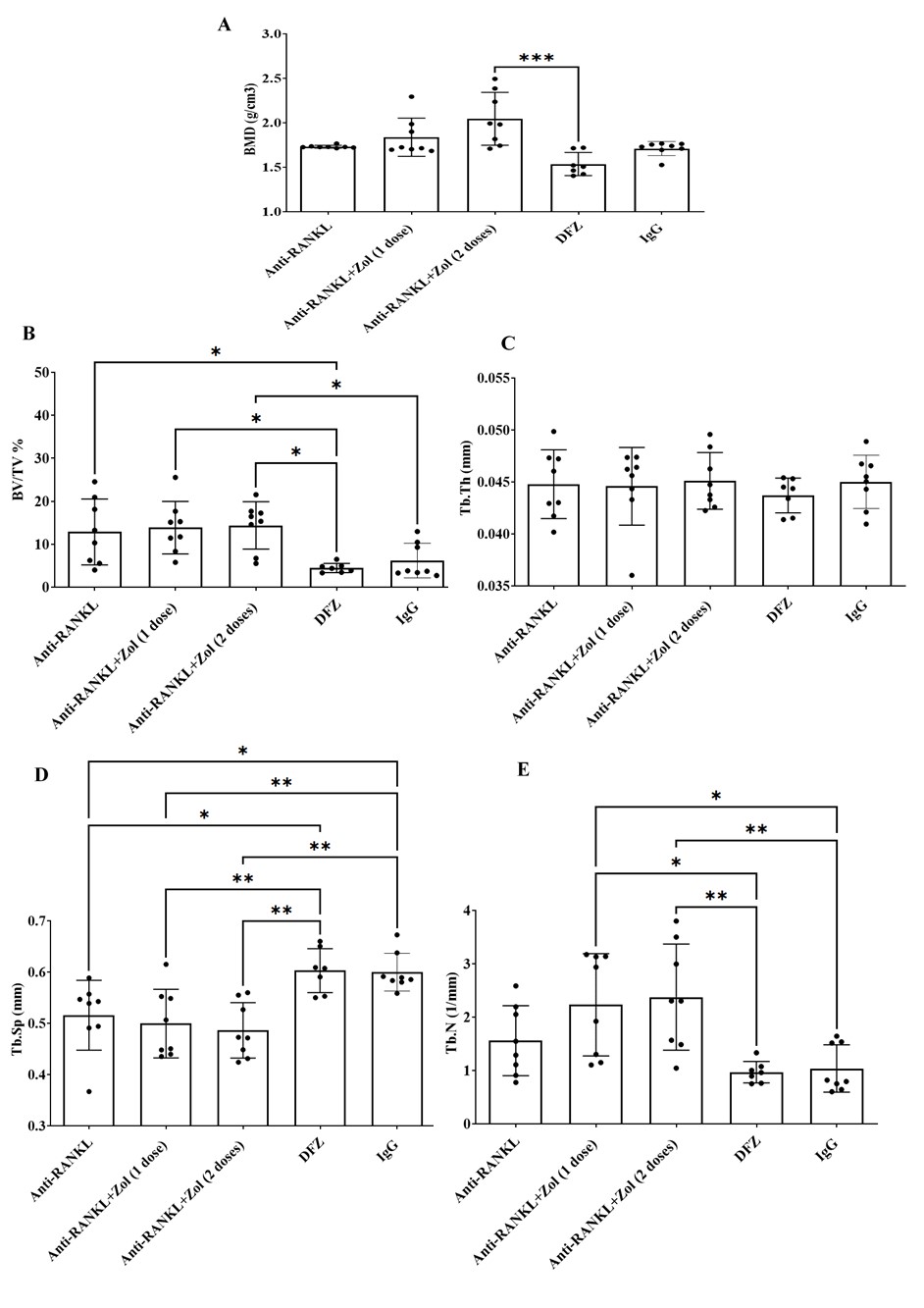


**Supp. Figure 3.** Cortical BMD (bone mineral density) of the tibia was significantly increased in anti-RANKL followed by 2 doses of Zol treated *mdx* mice compared to DFZ treated *mdx* mice (A). BV/TV (trabecular bone volume/tissue volume; %) and Tb. Sp. (trabecular separation; mm) were significantly increased and decreased, respectively in anti-RANKL, or anti-RANKL followed by 1 or 2 doses of Zol treated mdx mice compared to DFZ treated *mdx* mice (B,D). Tb. Th. (trabecular thickness; mm) was similar in all treatment groups (C). Tb. N. (trabecular number; mm^-1^) was significantly increased in anti-RANKL followed by 1 or 2 doses of Zol treated *mdx* mice compared to DFZ treated *mdx* mice (E). Data are represented as the means ± SEM. *p < 0.05; **p < 0.01; ***p < 0.001.

**Supp. Figure 4.** Fracture load of the tibia was significantly increased in anti-RANKL, or anti-RANKL followed by 1 or 2 doses of Zol treated *mdx* mice compared to DFZ or IgG treated *mdx* mice (B). The work to failure and yield loads were significantly increased in anti-RANKL followed by 1 or 2 doses of Zol treated *mdx* mice compared to DFZ treated *mdx* mice (C, F). Load at maximum stiffness was significantly increased in anti-RANKL followed by 2 doses of Zol treated *mdx* mice compared to DFZ treated *mdx* mice (E). Failure and work to fracture load were similar in all treatment groups (C). (A, D). Data are represented as the means ± SEM. *p < 0.05; **p < 0.01; ***p < 0.001.
